# Drug interaction in the emergency service

**DOI:** 10.1590/S1679-45082013000400010

**Published:** 2013

**Authors:** Meiry Fernanda Pinto Okuno, Raíssa Silveira Cintra, Cássia Regina Vancini-Campanharo, Ruth Ester Assayag Batista

**Affiliations:** 1 Universidade Federal de São Paulo, São Paulo, SP, Brazil

**Keywords:** Drug interactions, Drugs incompatibility, Patient safety

## Abstract

**Objective:**

To identify the occurrence of potential drug interactions in prescriptions for adult patients admitted to the Emergency Department of *Hospital São Paulo.*

**Methods:**

A cross-sectional and descriptive study. Its sample consisted of 200 medical prescriptions. The analysis of drug interactions was performed using the Drugs.com database, where they are classified according to severity of interaction as severe, moderate, mild and without interaction.

**Results:**

The number of drugs in prescriptions ranged from 2 to 19, and the average per prescription was 4.97 drugs. A total of 526 potential drug interactions were identified in 159 prescriptions (79.5%); in that, 109 were severe, 354 moderate, 63 mild interactions, and 41 showed no interaction.

**Conclusion:**

This study demonstrated potential drug interactions in 79.5% of prescriptions examined in the Emergency Department. Drug interactions can occur at any time when using medications and, during this working process, the nursing staff is involved in several steps. Therefore, training the nursing staff for the rational use of drugs can increase safety of care delivered to patients.

## RESUMO

**Objetivo:**

Identificar a ocorrência de potenciais interações medicamentosas em prescrições médicas de pacientes adultos internados no Serviço de Emergência do Hospital São Paulo.

**Métodos:**

Estudo de corte transversal e descritivo. A amostra foi composta por 200 prescrições médicas. A análise das interações medicamentosas foi realizada por meio da base de dados Drugs.com, onde são classificadas quanto à potencialidade de ação em interação grave, moderada, leve e sem interação.

**Resultados:**

O número de medicamentos das prescrições variou de 2 a 19, e a média por prescrição foi de 4,97 medicamentos. Foram identificadas 526 potenciais interações medicamentosas em 159 prescrições (79,5%); destas, 109 foram interações graves, 354 moderadas, 63 leves e 41 não apresentaram interação.

**Conclusão:**

Este estudo demonstrou potencial interação medicamentosa em 79,5% das prescrições analisadas no Serviço de Emergência. As interações medicamentosas podem acontecer em todos os momentos da utilização dos fármacos e, nesse processo de trabalho, a equipe de enfermagem está envolvida em diversas etapas. Portanto, instrumentalizar o enfermeiro para o uso racional dos fármacos pode aumentar a segurança da assistência ao paciente.

## INTRODUCTION

The last decades have witnessed an association between overcrowded emergency services and a decrease in the quality of care^(1)^.

In addition to overcrowding, other factors such as stress, insufficient number of healthcare professionals, high turnover of patients, and communication failure among multiprofessional teams turn emergency services into a critical site for the occurrence of problems^(2)^.

Some errors are associated with the use of medication: drug interactions, adverse events, allergic reactions, and medication errors^(3)^. The most frequently reported errors in emergency departments are associated with drug-related adverse events^(4)^.

Medical treatment is essential in healthcare. Yet, it may cause disease and death, and become an economic burden to society^(3)^. Despite the fact that the simultaneous use of several drugs frequently increases their therapeutic efficacy, certain combinations are harmful and may increase the risk of drug interaction^(5)^.

In emergency departments, often several drugs are prescribed simultaneously. This may cause non-therapeutic interaction in a patient and eventually harm the patient^(6)^.

Drug interaction is a pharmacological response where the effect of one or more drugs is changed by their simultaneous administration, or by previous use of other drugs^(5)^. Drug interactions depend on a number of variables, such as clinical condition of the patient, number and characteristics of drugs, and are aggravated by the fact that health professionals are unaware of the actions of these medicines. The nurses are in charge of preparing prescriptions, and many times potential interactions are disregarded^(7)^. Drug interaction is one of the most important variables in the treatment, and its clinical significance is difficult to predict^(8)^.

The clinical pharmacists play several roles in medical therapy, such as revising prescriptions to investigate drug interactions and writing recommendations, which must contribute to patient's safety^(9)^.

In emergency departments, nurses and all members of the multiprofessional team must offer immediate care to critical patients, and use their clinical judgement regarding the use of prescribed drugs, in order to avoid possible drug interaction or decreased efficacy in the treatment proposed^(7)^.

## OBJECTIVE

To identify the occurrence of potential drug interactions in prescriptions for adult patients admitted to the Emergency Department *of Hospital São Paulo.*

## METHODS

This article describes a cross-sectional study carried out at *Hospital São Paulo.* The convenience sample consisted of all medical prescriptions within the first 24 hours of adult patients admitted to the Clinical Emergency Room of the Emergency Department, from March to July 2012.

Data collection consisted in transcribing all pieces of information to individual patient files. The following variables were recorded: date of admission, gender, age, past medical/surgical history, name of the drug, dosage, pharmaceutical formulation and route of administration.

The analysis of drug interaction was performed using the database Drugs.com. In the database, all drugs in the prescription were matched and a list with the drug interactions was obtained, classifying them as to severity as severe, moderate, and mild interaction, and no interaction^(10)^.

Data was analyzed by means of descriptive statistics and presented as graphs and tables.

The study was conducted after the analysis and approval by the Research Ethics Committee of *Universidade Federal de São Paulo* (CAAE: 00849512.6.0000.5505).

## RESULTS

The sample comprised 200 medical prescriptions, and 53.5% (n=103) of them had been prescribed to women with mean age of 51.3 years.

The most frequent conditions described in patients' past history were cardiovascular (66%) and endocrine (25%) disorders.

The number of drugs listed in the prescriptions varied from 2 to 19; in average, 4.97 drugs were listed per prescription. A total of 779 drugs had been prescribed. Metoclopramide (28.1%), sodium dipyrone (15.0%), tramadol hydrochloride (10.0%), omeprazole (9.5%), phenytoin (8.0%) and clarithromycin (7.4%) were the most frequently prescribed drugs.

**Figure 1:**
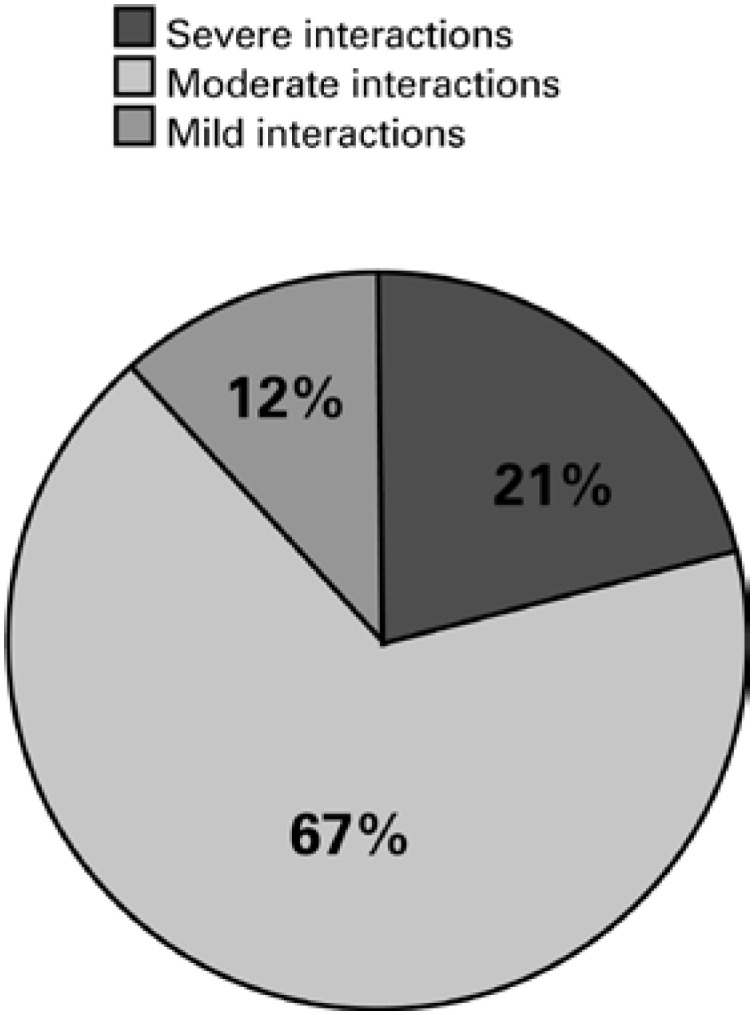
Potential drug interactions in the prescriptions of patients admitted to the Clinical Emergency Service of *Hospital São Paulo,* between March and July, São Paulo, 2012

The analyses carried out using the database Drugs. com identified 526 potential drug interactions in 159 prescriptions (79.5%); in that, 109 (21%) were severe; 354 (67%) moderate; 63 (12%) mild interactions; and 41 prescriptions had no drug interaction (Figure 1).

Table 1 lists the drug interactions classified as severe according to the database Drugs.com.

**Table 1:** Frequency of severe drug interactions identified in the prescriptions of patients admitted to the Clinical Emergency Service of *Hospital São Paulo,* between March and July, São Paulo, 2012

Drug I	Drug II	n	Frequency (%)
Metoclopramide	Tramadol	33	30.4
Sodium dipyrone	Enoxaparin sodium	13	11.9
Warfarin	Enoxaparin sodium	11	10.2
Amiodarone	Clarithromycin	6	5.6
Tramadol	Meropenem	5	4.6
Ciprofloxacin	Tramadol	3	2.7
Haloperidol	Fluconazole	3	2.7
Haloperidol	Metoclopramide	3	2.7
Morphine	Tramadol	3	2.7
Amiodarone	Furosemide	2	1.8
Amiodarone	Haloperidol	2	1.8
Acetylsalicylic acid	Enoxaparin sodium	2	1.8
Warfarin	Clarithromycin	2	1.8
Other*	Other	21	19.3
Total		109	100

* Frequencies <1.8% were included under the category others.

Table 2 lists the drug interactions classified as moderate according to the database Drugs.com.

**Table 2:** Frequency of moderate drug interactions identified in the prescriptions of patients admitted to the Clinical Emergency Service of *Hospital São Paulo,* between March and July, São Paulo, 2012

Drug I	Drug II	n	Frequency (%)
Phenytoin	Omeprazole	24	6.8
Captopril	Heparin	14	3.9
Captopril	Enoxaparin sodium	8	2.3
Warfarin	Tramadol	8	2.3
Other*	Other	300	84.7
Total		354	100

* Frequencies <1.8% were included in the category others.

Table 3 lists drug interactions that were considered mild according to the database Drugs.com.

**Table 3:** Frequency of mild drug interactions identified in the prescriptions of patients admitted to the Clinical Emergency Service of *Hospital São Paulo,* between March and July, São Paulo, 2012

Drug I	Drug II	n	Frequency (%)
Clarithromycin	Omeprazole	7	11.1
Dipyrone Sodium	Heparin	5	7.9
Ceftriaxone Sodium	Heparin	5	7.9
Acetylsalicylic acid	Omeprazole	4	6.3
Oxacillin	Heparin	4	6.3
Rifampicin	Midazolam	4	6.3
Ciprofloxacin	Metoclopramide	3	4.8
Metoclopramide	Morphine	3	4.8
Enalapril	Amlopidine	2	3.2
Furosemide	Acetylsalicylic acid	2	3.2
Nifedipine	Omeprazole	2	3.2
Levothyroxine	Simvastatin	2	3.2
Ranitidine	Ketoprofen	2	3.2
Others*	Others	18	28.6
Total		63	100

* Frequencies <1.8% were included in the category others.

## DISCUSSION

This study points out the need to assess possible drug interactions in prescriptions made in the Emergency Services. The risk associated with drug interactions was shown by the number of potential severe and moderate drug interactions identified in prescriptions written during the first 24 hours of admission to the service.

In this study, the mean age of patients was 51.3 years. This result is similar to that of the study carried out at the Intensive Care Unit (ICU), in which the mean age of patients was 56.3^(11)^.

Most frequently reported conditions were cardiovascular diseases (66%) and *diabetes mellitus* (25%). These results are also in line with the findings of another study that described the prevalence of potential drug interactions in clinical and surgical units where cardiovascular (42,5%) and endocrine diseases (20.8%) were most frequently observed^(12)^. Further studies on this subject are required, due to the limited number of investigations on presence of comorbidities and potential risks of drug interactions. This study showed a high prevalence of potential drug interactions (79.5%) among the drugs found in the prescriptions. This is corroborated by the findings of another study carried out in ICU (67.1%), which was used to compare data because there is a lack of studies in the area of emergency services^(13)^.

The number of drugs listed in the prescriptions varied between 2 and 19, and the mean number of drugs per prescription was 4.97. Another study that evaluated potential drug interactions in prescriptions for patients in Intensive Care Units (ICUs) and Rooming-in identified a larger number of drugs (5 to 22), with an average of 10.9 drugs per prescription^(14)^.

Assessments carried out using the database Drugs. com, showed a high prevalence of interactions at the Emergency Department. Out of these, 109 were severe, 354 moderate and 63 mild cases. These results are greater than those of another study that identified 38 prescriptions for patients at the ICU – in that, 105 were severe, 171 moderate and 18 mild^(14)^ interactions.

Most commonly prescribed drugs associated with moderate and mild interactions were phenytoin, omeprazole, captopril, sodium heparin, clarithromycin and sodium dipyrone. These interactions may worsen the clinical condition of the patient, require additional treatment, increase the hospital length of stay, or cause discomfort. These results are consistent with another study, which also found out that dipyrone sodium, captopril and sodium heparin were the drugs most commonly involved in drug interactions^(13)^.

Interaction between tramadol and metoclopramide was the most frequent (30.27%) among severe drug interactions. Similar information was reported in another study where 60% of drug interactions included these two drugs^(14)^. The assessment carried out on the database Drugs.com reveals that this combination is highly lethal, and depending on the dose may cause death of the patient^(10)^.

The risk of seizures increases when tramadol is simultaneously administered with other medications, such as selective serotonin reuptake inhibitors, aminooxidase inhibitors, neuroleptic agents that stimulate the nervous system, opioids, among others. When administered alone, these agents are often epileptogenic and may have potentiated effects when given in combination with other drugs^(10)^.

In addition to interaction with metoclopramide, tramadol presented potential interaction with other drugs, such as antibacterial agents and drugs that act on the central nervous system, such as amitriptyline, fluoxetine and morphine^(14)^.

In the case of moderate interactions, the combined use of omeprazole and phenytoin was the most frequent (10.6%) and, despite their different indications, these medications have antagonistic effects. Similar information was reported in a study that evidenced this interaction in 29.3% of findings^(15)^.

Omeprazole may increase the serum concentration of phenytoin and thus its toxicity, inhibiting liver metabolism and drug excretion. Furthermore it can interact in a similar fashion with other hydantoins. This medication is more susceptible to potential interactions when given at doses >40mg/day^(16)^. In addition to laboratory follow-up to assess the side effects of phenytoin, the combined use of these medications requires observation of signs and symptoms that indicate toxicity: sleepiness, visual impairment, changes in metal status, seizures, nausea and ataxia^(10)^.

Mild interactions do not present significant clinical value because they do not potentially alter the effects of the drugs^(14)^. Out of the 63 mild interactions, 11.1% were associated with the use of omeprazole and clarithromycin. A similar result was described in a study on factors associated with medical prescription of antibiotics in public pharmacies, which presented 16.6% of this type of interaction^(17)^.

Clarithromycin may increase and extend the serum concentration of omeprazole in the body. This may be associated with the fact that clarithromycin inhibits cytochrome in liver enzymes, which are involved in omeprazole metabolism. Combined administration of omeprazole may also cause an increase in serum clarithromycin concentration. This increase could be associated with the effect of omeprazole on gastric pH(10).

Another important concern is the presence of more than one drug interaction on the same prescription. Drug interactions must be investigated in these cases, even in the absence of immediate clinical investigation, because an early suspicion would enable the health team to prepare for possible pharmacodynamic or pharmacokinetic adverse reactions^(18)^.

## CONCLUSIONS

This study demonstrated potential drug interaction in the majority of prescriptions assessed in the Clinical Emergency Sector of *Hospital São Paulo.* Due to the characteristics of emergency services, a number of strategies should be implemented to increase patient safety.

Further studies must be conducted to cover all phases of drug use, and include the design of protocols to ensure greater safety in the use of drugs in these units.
